# Effects of continuous and interval training on inflammatory and vascular adhesion markers in sedentary individuals with type 1 diabetes

**DOI:** 10.1007/s00421-025-05983-z

**Published:** 2025-09-19

**Authors:** Alberto Hermo-Argibay, Laura Brugnara, Serafín Murillo, Joan-Marc Servitja, Víctor M. Víctor, Anna Novials, Susana Rovira-Llopis

**Affiliations:** 1 Service of Endocrinology and Nutrition, Foundation for the Promotion of Health and Biomedical Research in the Valencian Region (FISABIO), University Hospital Doctor Peset, Valencia, Spain; 2 Institut d‘Investigacions Biomèdiques August Pi I Sunyer (IDIBAPS), Barcelona, Spain; 3 Centro de Investigación Biomédica en Red de Diabetes y Enfermedades Metabólicas Asociadas (CIBERDEM), Instituto de Salud Carlos III, Madrid, Spain; 4 Department of Endocrinology, Institut de Recerca Sant Joan de Déu, Hospital Sant Joan de Déu, Barcelona, Spain; 5 Department of Physiology, University of Valencia, INCLIVA (Biomedical Research Institute Valencia), Valencia, Spain; 6 CIBERehd - Department of Pharmacology, University of Valencia, Valencia, Spain

**Keywords:** Type 1 diabetes, Exercise, Inflammation, Cytokines, Adhesion molecules

## Abstract

**Purpose:**

This study evaluates and compares the effects of continuous and interval training on inflammatory and adhesion molecules in subjects with Type 1 diabetes mellitus (T1D) and healthy controls.

**Methods:**

Using Luminex X-MAP, serum inflammatory and adhesion molecules were measured in 50 non-obese, sedentary adults (78% women; mean age 34 years), including 27 with T1D and 23 healthy controls.

**Results:**

Subjects with T1D exhibited a tendency towards decreased interleukin 10 (IL-10) levels and increased intercellular adhesion molecule (ICAM1) levels compared to controls. Exercise training, specifically high-intensity interval training (HIIT), increased IL-10 levels in the T1D group. Among controls, we observed a decrease in vascular cell adhesion molecule 1 (VCAM1) after continuous training, and in platelet selectin (P-selectin) after HIIT. The correlation studies revealed that subjects with higher baseline maximal oxygen uptake (VO_2_max) achieved greater reductions in P-selectin levels with training and that levels of VCAM1 were further reduced by training in subjects with higher baseline metabolic equivalents (METS).

**Conclusion:**

Our findings show that the effects of exercise on inflammatory cytokines and adhesion molecules vary depending on the training modality and the population studied. Additionally, our data suggest that physical activity and fitness levels influence individual responses to exercise in relation to adhesion molecules in healthy and subjects with T1D.

## Introduction

In type 1 diabetes mellitus (T1D), β-cells within the pancreatic islets of Langerhans are destroyed, resulting in the loss of insulin secretion (Delmastro and Piganelli 2011). This process is mediated by activation of the c-Jun N-terminal kinase (JNK) pathway by pro-inflammatory cytokines, which contributes to β-cell destruction (Kaneto et al. 2005). Additionally, β-cells secrete cytokines such as interleukin-1 beta (IL-1β), which, under normal conditions, promotes their proliferation (Maedler et al. 2002). However, in T1D, in conjunction with tumor necrosis factor-alpha (TNFα), IL-1β can induce apoptosis (Cnop et al. 2005). In addition, it has been demonstrated that TNFα and interleukin-6 (IL-6), both pro-inflammatory cytokines, play crucial roles in T1D progression and the regulation of insulin secretion (Atri et al. 2018).

Chronic low-grade inflammation and sustained hyperglycemia promote endothelial dysfunction, thereby disrupting vascular tone regulation and homeostasis (D.-R. Yang et al. 2024). This dysfunction leads to an imbalance that favors vasoconstriction and poor regulation of vascular smooth muscle (Bertoluci et al. 2008), contributing to the vascular complications that are a leading cause of morbidity and mortality in patients with T1D (James et al. 2014). Endothelial dysfunction also promotes atherogenesis and the development of subclinical atherosclerosis by activating signaling pathways in endothelial cells that increase the production of cellular adhesion molecules (CAMs), cytokines, and chemokines, resulting in the interaction of leukocytes with the vascular wall, to which they subsequently adhere. This process is mediated by selectins. In inflammatory processes, leukocyte recruitment begins when the movement of leukocytes through the blood flow slows and they begin to roll along the endothelial walls, eventually adhering to them and migrating through the endothelium towards the focus of infection (Leick et al. 2014). In this sense, a recent study by Canet et al. (2022) demonstrated that patients with T1D exhibit increased leukocyte–endothelial interactions, with a reduced rolling velocity over the vascular wall and a higher number of adhered leukocytes compared to a control group.

Maintaining high levels of physical activity is reported to benefit the quality of life of patients with T1D. Notably, it improves glucose metabolism regulation by increasing time in range and reducing fasting glucose levels (Riddell et al. 2023), as well as enhancing insulin sensitivity, lowering glycated hemoglobin, and improving lipid profiles (Salem et al. 2010). Additionally, it enhances metabolic flexibility and cardiorespiratory fitness (D ‘ hooge et al. 2011; Heyman et al. 2007; L. Roberts et al. 2002). These benefits indicate a lower risk of developing the cardiovascular diseases associated with diabetes (Wu et al. 2019). It is noteworthy that in T1D, muscle carnosine levels are increased and associated with a more atherogenic lipoprotein profile, which is especially relevant in subjects with T1D and low cardiovascular fitness (Brugnara et al. 2022). These findings highlight the beneficial effects of physical exercise and physical fitness status on vascular health and cardiometabolic risk factors.

Exercise also exerts an anti-inflammatory effect, such as that produced in muscle contractions in which IL-6 is released, which stimulates the production of anti-inflammatory cytokines, thus inhibiting TNFα activity and reducing inflammation (Petersen & Pedersen 2005). Notably, interleukin-10 (IL-10), the natural receptor antagonist IL-1Ra, interleukin-1 alpha (IL-1α), IL-1β, and interleukin-8 (IL-8) are involved in this anti-inflammatory response, with a potential inhibition of T-cell activation and nuclear factor kappa B (NF-κB) (Sharifinejad et al. 2022). Beneficial effects are also observed on adhesion molecules such as CAMs, resulting in reductions in intercellular adhesion molecule 1 (ICAM1), vascular cell adhesion molecule 1 (VCAM1), and platelet-selectin (P-selectin) levels (J. Yang et al. 2017).

In clinical research involving continuous aerobic exercise, the majority of interventions have traditionally been categorized as moderate-intensity continuous training (MICT) (Gao et al. 2025). However, according to contemporary intensity domain classifications, many of these protocols actually fall within the heavy-intensity domain, often slightly above the first lactate threshold (Iannetta et al. 2020). Therefore, it may be more appropriate to refer to them as heavy-domain intensity continuous training (HICT). This reclassification highlights the need for greater precision when describing exercise intensity domains in the scientific literature. In parallel, within high-intensity interval training (HIIT) interventions, a wide variety of modalities has been investigated. The most common approaches involve intervals lasting 30 s to 2 min, performed at intensities ranging from 85 to 110% of the peak power output (PPO) attained during a maximal oxygen uptake (VO₂max) test, or at 85–95% of maximal heart rate (Gao et al. 2025; Tschakert and Hofmann 2013).

HIIT has been gaining increasing attention in exercise intervention studies, positioning itself as an effective strategy for improving aerobic capacity (Opazo-Díaz et al. 2024). Despite numerous studies in the literature, it remains unclear whether interval and/or continuous training exert beneficial effects on circulating cardiovascular disease markers in healthy subjects and individuals with T1D, such as pro- and anti-inflammatory cytokines and CAMs, particularly because individuals engaging in the same level of physical activity show differences in physical capacities (Murillo et al. 2024). In the present study, we have explored differential training-induced effects on inflammatory cytokines and CAMs depending on the type of training (interval/continuous) and the target population (healthy/T1D).

## Methods

### Study design

Almost all participants in this study were selected from a cohort of a previous study by Murillo et al. (2022). Training sessions took place in the laboratory of Exercise and Diabetes at IDIBAPS-Hospital Clínic Barcelona, where blood samples were also collected. These samples were subsequently processed at the Translational Research Unit of Endocrinology and Metabolism of the University Hospital Doctor Peset.

### Participants

The present study included 50 sedentary participants, 27 of whom had T1D. Inclusion criteria for the T1D group required a diagnosis of at least 6 months, an age between 18 and 50 years, hemoglobin A1c below 9.5%, and no regular physical exercise in the preceding 3 months. Participants with T1D had a mean time since diagnosis of 18.8 ± 9.7 years. The exclusion criteria included advanced diabetic complications (with the exception of non-proliferative retinopathy), musculoskeletal injuries, pregnancy, or any condition limiting the ability to perform physical exercise. The remaining 23 participants were healthy individuals, with an effort made to ensure a similar age and sex distribution to that of the T1D group.

### Measurements

VO₂max and PPO were determined using a ramp incremental test on a cycle ergometer. The test began with a 5-min warm-up at low intensity (40 watts), followed by continuous increments of 15 watts every minute until volitional exhaustion. Participants were instructed to maintain a cadence between 60 and 80 revolutions per minute. Maximum strength was assessed via a one-repetition maximum leg press test. Body composition was evaluated using an iDXA Lunas (GE Healthcare). Blood samples were collected for analysis. All initial measurements were repeated 48 to 72 h after the last training session. Blood samples were sent to the Endocrinology and Nutrition Translational Research Unit at University Hospital Doctor Peset (Valencia, Spain) for analysis. The physical activity level and the Metabolic Equivalents of Task (METs) of the subjects have been determined using the International Physical Activity Questionnaire (IPAQ).

### Training protocol

The exercise program consisted of three sessions per week on alternate days over 3 weeks (a total of nine sessions), with each group following a specific exercise regimen performed on a cycle ergometer (Wattbike PRO, Nottingham, UK). The HICT group engaged in continuous training within the heavy-domain intensity for 30 min at 65–70% of VO₂max. The HIIT group performed high-intensity interval training, consisting of 10 repetitions of 30 s at 100–120% of PPO, interspersed with 2-min intervals of active recovery at 40–50% VO₂max. Both regimens included a 5-min warm-up and a 5-min cool-down.

### Immunoassays

Serum levels of inflammatory mediators, IL-6, IL-1β, and TNFα, anti-inflammatory mediators such as IL-10, as well as those of myeloperoxidase (MPO) and the adhesion molecules VCAM1, ICAM1, and P-Selectin, were quantified using Luminex® with xMAP technology (Luminex®200, Luminex Corporation™, Austin, TX, USA). Milliplex® kits (Merck KGaA, Darmstadt, Germany), specifically the “Human Cardiovascular Disease Magnetic Bead Panel 2” and “Human High Sensitivity T Cell Magnetic Bead Panel,” were used for quantification. The analyses were performed in duplicate, with intra- and inter-assay coefficients of variation of < 5.0% and < 15%, respectively.

### Statistical analysis and data representation

Data analysis was conducted using SPSS® 20.0 (IBM SPSS™ Statistics, Chicago, USA). The Shapiro–Wilk test was employed to check the normality of population distribution. Parametric data are presented as mean ± standard deviation. Qualitative data are expressed as percentages. A student’s t-test was used for comparing parametric data between independent groups (control vs. T1D) and paired t-tests were employed for dependent groups (pre- vs. post-intervention). Bivariate Pearson correlation was used for correlation analysis. A 95% confidence interval was applied for all tests, and differences were considered significant at *p* < 0.05. A tendency was assumed for *p* values between 0.1 and 0.05. Data were analyzed and represented using GraphPad Prism® v.9.0.2 (GraphPad Software™, Boston, MA, USA).

## Results

### Cohort characteristics

In the present study, we assessed a total of 50 sedentary subjects comprised of patients with T1D (27) and healthy controls (23). There were no significant differences between the groups regarding anthropometric parameters and body composition at the start of the exercise intervention (Table 1). The following fitness-related parameters were also similar between controls and subjects with T1D: VO_2_max, METs, external load relative to VO_2_max and maximum weight lifted in the leg press.

**Table 1:** Anthropometric and fitness-related parameters and serum levels of cytokines and adhesion molecules in the study population at baseline

	Control	T1D	*p*-value
Sample size	**23**	**27**	**–**
Sex (% women)	86.9%	70.4%	–
Age (years)	31.5 ± 6.9	35.9 ± 9.3	0.071
Body mass (kg)	63.9 ± 12.1	70.3 ± 14.3	0.112
BMI (kg/m^2^)	23.0 ± 3.5	25.2 ± 4.3	0.07
Fat (%)	31.7 ± 7.1	32.7 ± 7.4	0.667
Lean mass (kg)	41.7 ± 7.64	44.9 ± 8.9	0.203
Visceral adipose tissue (g)	290.3 ± 316.6	444.8 ± 441.9	0.203
METs	1048 ± 726	794 ± 702	0.820
VO_2_max (ml·kg·min)	23.7 ± 7.0	23.7 ± 6.0	0.999
Peak power output relative to body mass (W/kg BM)	2.1 ± 0.6	2.0 ± 0.6	0.391
Maximum weight lifted in leg press relative to body mas (kg/kg BM)	2.29 ± 0.53	2.13 ± 0.77	0.473
ICAM1 (ng/mL)	69.59 ± 14.72	79.71 ± 21.57	0.059
MPO (ng/mL)	204.4 ± 141.8	210.5 ± 148.1	0.771
P-selectin (ng/mL)	117.94 ± 42.25	117.13 ± 51.26	0.922
VCAM1 (ng/mL)	966.3 ± 176.0	967.3 ± 214.5	0.933
IL-10 (pg/mL)	20.00 ± 14.73	13.38 ± 8.89	0.078
IL-1β(pg/mL)	2.99 ± 1.63	2.84 ± 1.37	0.659
IL-6 (pg/mL)	2.70 ± 1.54	2.68 ± 1.44	0.703
TNFα (pg/mL)	12.54 ± 5.30	12.50 ± 2.51	0.905

The results are expressed as mean ± standard deviation or as a percentage.

*BMI* body mass index, *BM* body mass, *ICAM1 *intercellular adhesion molecule 1, *IL* interleukin, *METs* metabolic equivalents, *MPO* myeloperoxidase, *TNFα* tumor necrosis factor alpha, *VCAM1* vascular cell adhesion molecule 1, *VO*_*2*_*max* maximal oxygen uptake

After the 3-week exercise intervention, significant changes were observed in the subjects’ anthropometric and fitness characteristics (Table 2). In short, HIIT increased relative strength in both groups, improved VO_2_max and PPO relative to body mass (W/kg) in healthy subjects and decreased total fat and increased lean mass in T1D, whereas HICT increased PPO relative to body mass (W/kg) in both groups and increased relative strength among subjects with T1D.

**Table 2:** Effect of the exercise intervention according to the group and protocol used

	Control						T1D					
	HIIT			HICT			HIIT			HICT		
	Pre	Post	*P* value	Pre	Post	*P* value	Pre	Post	*P* value	Pre	Post	*P* value
Body mass (kg)	66.8 ± 13.0	63.0 ± 18.1	ns	64.0 ± 12.7	63.9 ± 12.8	ns	72.0 ± 16.2	72.2 ± 16.2	ns	68.1 ± 12.5	68.8 ± 12.2	ns
BMI (kg/m)	23.2 ± 3.9	19.83 ± 9.13	ns	23.4 ± 3.4	23.3 ± 3.4	ns	25.3 ± 4.8	25.4 ± 4.9	ns	25.4 ± 4.4	25.6 ± 4.1	ns
Fat (%)	30.9 ± 8.7	29.5 ± 8.9	ns	32.7 ± 5.0	32.3 ± 4.9	ns	32.4 ± 8.1	31.7 ± 8.0	**0.006**	34.0 ± 6.9	34.0 ± 7.0	ns
Lean mass (kg)	42.2 ± 6.4	42.8 ± 6.5	ns	43.0 ± 9.70	43.26 ± 9.68	ns	45.5 ± 9.8	46.1 ± 9.8	**0.01**	42.9 ± 6.6	43.4 ± 6.6	ns
Visceral fat (g)	306.0 ± 386.9	317.5 ± 373.8	ns	268.1 ± 238.5	289.6 ± 249.8	ns	533.7 ± 510.0	526.8 ± 515.7	ns	383.1 ± 341.7	377.0 ± 351.1	ns
Vo_2_max (ml/kg/min)	24.2 ± 8.4	27.9 ± 6.7	**0.002**	23.9 ± 4.1	24.6 ± 4.7	ns	23.62 ± 5.62	24.7 ± 5.0	ns	23.8 ± 6.9	24.3 ± 4.8	ns
Peak power output relative to body mass (W/kg)	2.28 ± 0.72	2.49 ± 0.71	**0.017**	1.75 ± 0.29	2.15 ± 0.33	**0.001**	2.19 ± 0.58	2.29 ± 0.48	ns	1.49 ± 0.34	1.67 ± 0.37	**0.004**
Relative strength (kg/kg body weight)	1.93 ± 0.30	2.24 ± 0.45	**0.019**	2.49 ± 0.95	2.79 ± 1.20	ns	2.09 ± 0.31	2.28 ± 0.34	**0.023**	2.16 ± 0.75	2.32 ± 0.83	**0.013**

The result is expressed as mean ± standard deviation. ns *P* value > 0.05 when compared using a paired samples t-test

Data is presented as mean ± standard deviation

BMI body mass index, VO_*2*_max maximal oxygen uptake*. P* values indicates significant differences versus baseline

### Inflammation and adhesion molecules

To analyze the inflammatory status of the subjects and the impact of exercise on this condition, we quantified the presence of key molecules involved in the inflammatory response: IL-10, an anti-inflammatory marker, and the pro-inflammatory cytokines IL-1β, IL-6, and TNFα. A comparison at pre-intervention between patients with T1D and controls showed a trend toward lower levels of IL-10 in patients with T1D (mean difference of 6.62 ± 3.67, *p* = 0.078) (Table 1). No significant differences were observed in the levels of IL-1β, IL-6, and TNFα between T1D and controls.

**Fig. 1:**
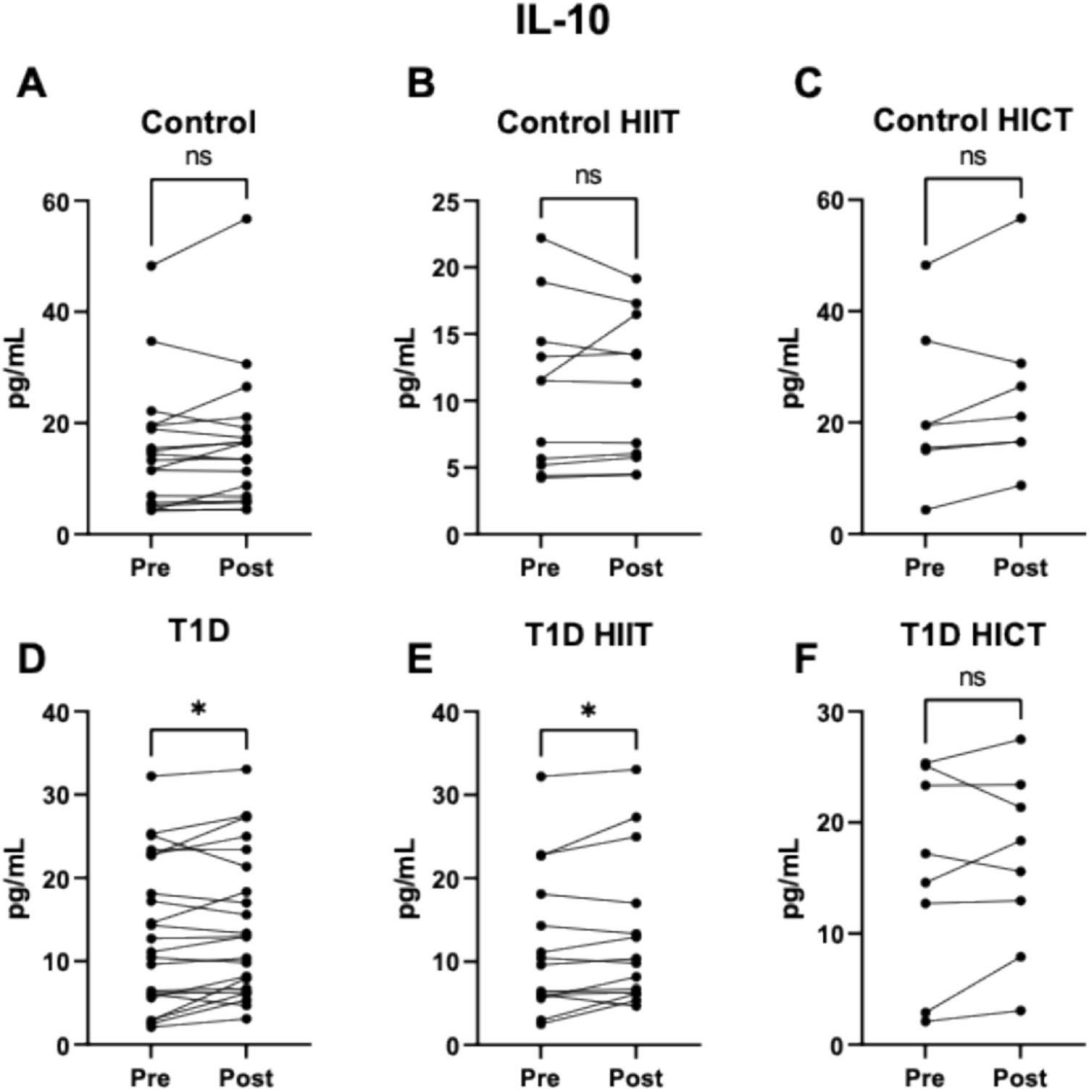
Effect of exercise on the anti-inflammatory molecule IL-10 in: **A**. Control group (both exercise protocols). **B**. Control group HIIT protocol. **C**. Control group HICT protocol. **D**. T1D Group (both exercise protocols). **E**. T1D group HIIT protocol. **F**. T1D group HICT protocol. ns *p* > 0.05. * *p* < 0.05 when compared using a paired Student’s t-test. Abbreviations: IL-10: Interleukin 10. T1D: Type 1 Diabetes. HICT: Heavy-domain Intensity Continuous Training. HIIT: High-Intensity Interval Training. Preference for color: online only

Upon analyzing the impact of training, we did not detect significant changes in the control population in terms of IL-10 levels in response to HIIT or HICT training (Fig. 1A–C). However, a significant increase in IL-10 levels was observed in patients with T1D (7.5% increase; *p* = 0.042) (Fig. 1D). When analyzed by training modality, this increase was specifically observed in the T1D group that underwent the HIIT program (8.8% increase; *p* = 0.044) (Fig. 1E), while no significant change was detected in the T1D group following HICT (Fig. 1F). These results indicate that changes in IL-10 levels occurred only in response to HIIT, not to continuous training.

To evaluate the potential impact of exercise on the initial stages of atherosclerosis, we quantified circulating levels of molecules involved in leukocyte-endothelium interactions. Specifically, we measured P-selectin (which facilitates leukocyte attraction to the endothelium), MPO (a pro-oxidant enzyme released by leukocytes), and the adhesion molecules ICAM1 and VCAM1 (which mediate leukocyte rolling, adhesion, and migration). A comparison between the two pre-intervention groups revealed a trend toward higher ICAM1 levels in patients with T1D (mean 69.59 ± 14.72 control group vs mean 79.71 ± 21.57 T1D group, ng/mL, *p* = 0.059) (Table 1).

**Fig. 2:**
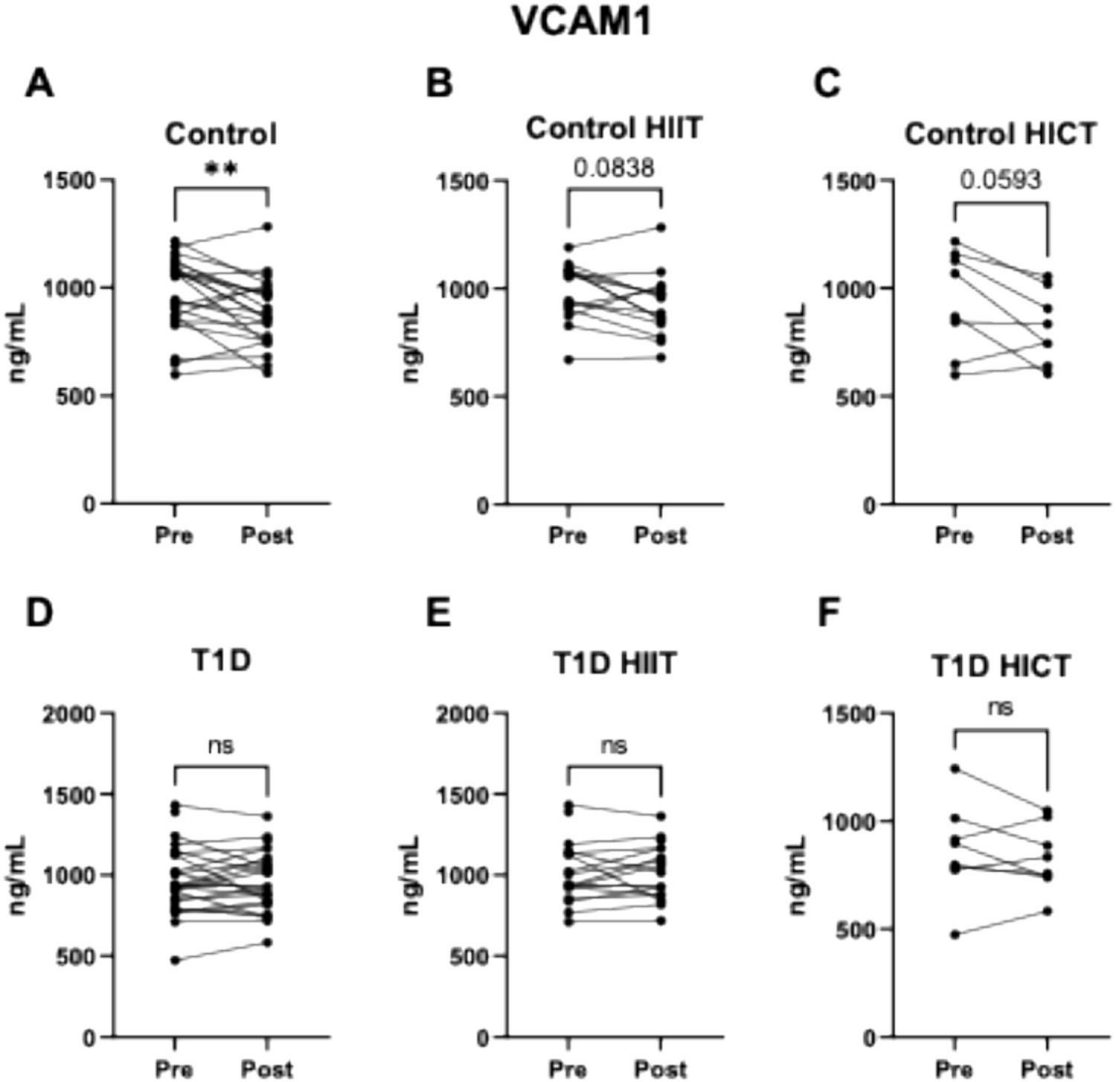
Effect of exercise on the adhesion molecule VCAM1 in: **A**. Control group (both exercise protocols). **B**. Control group HIIT protocol. **C**. Control group HICT protocol. **D**. T1D Group (both exercise protocols). **E**. T1D group HIIT protocol. **F**. T1D group HICT protocol. ns *p* > 0.05. # indicates a trend (*p* > 0.05 and < 0.1). * *p* < 0.05. ** *p* < 0.01 when compared using a paired Student’s t-test. Abbreviations: *IL-10* Interleukin 10, *T1D* Type 1 Diabetes, *HICT* Heavy-domain Intensity Continuous Training, *HIIT* High-Intensity Interval Training. Preference for color: online only

A significant reduction in VCAM1 levels was observed in the control group (8.4% decrease; *p* = 0.009) (Fig. 2A). When analyzed by training modality, reductions were observed in both subgroups, although these did not reach statistical significance: 5.8% in the HIIT group (*p* = 0.0838) and 13.1% in the HICT group (*p* = 0.0593) (Fig. 2B–C). No significant changes in VCAM1 levels were detected in participants with T1D, regardless of training modality (Fig. 2D–F).

**Fig. 3:**
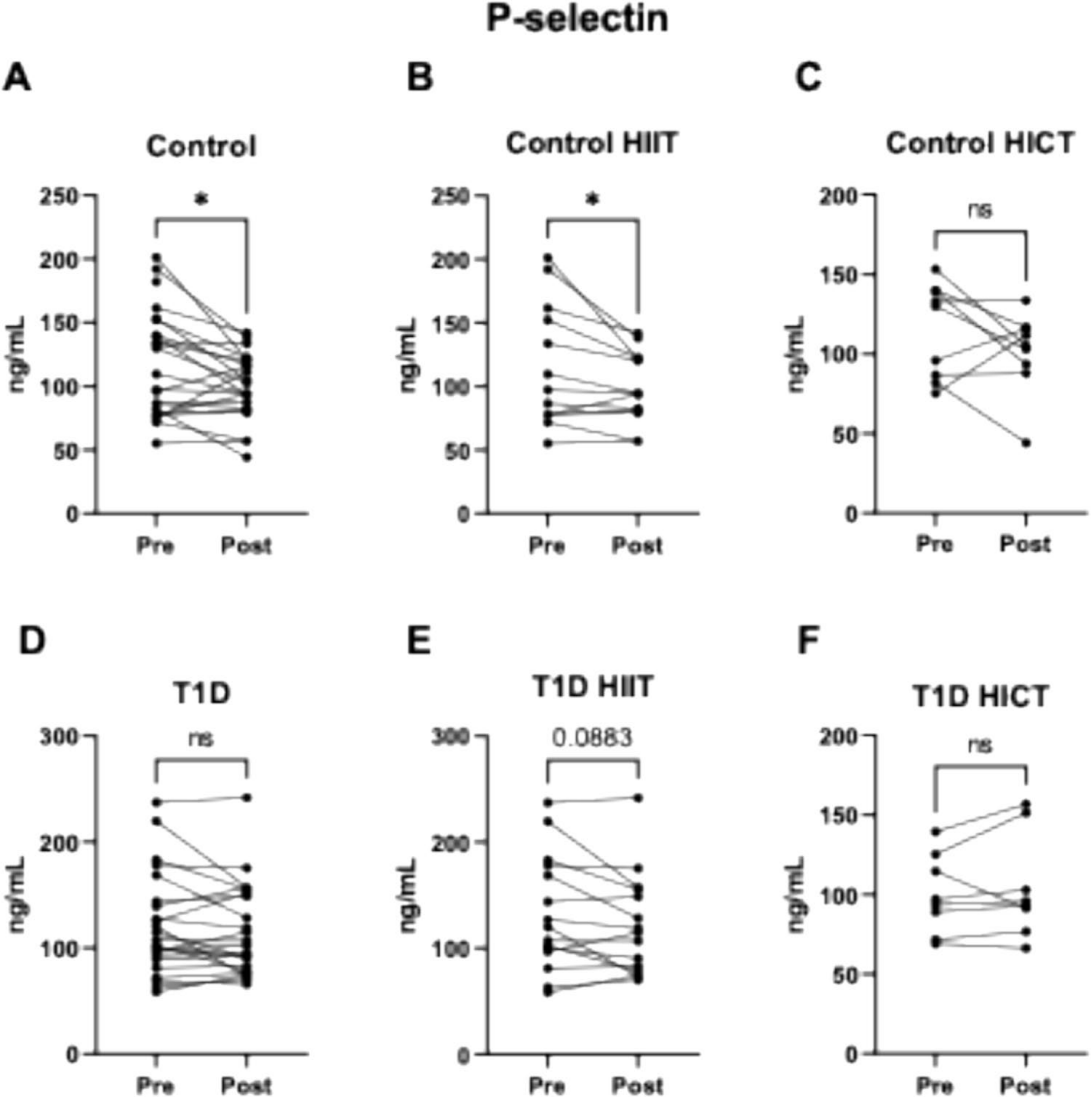
Effect of exercise on the adhesion molecule P-selectin in: **A** Control group (both exercise protocols). **B** Control group HIIT protocol. **C** Control group HICT protocol. **D** T1D Group (both exercise protocols). **E** T1D group HIIT protocol. **F** T1D group HICT protocol. ns *p* > 0.05. # indicates a trend (*p* > 0.05and < 0.1)* *p* < 0.05. ** *p* < 0.01 when compared using a paired Student’s t-test. Abbreviations: *IL-10* Interleukin 10, *T1D* Type 1 Diabetes, *HICT* Heavy-domain Intensity Continuous Training, *HIIT* High-Intensity Interval Training. Preference for color: online only

Regarding P-selectin, a significant reduction was observed in the control group (13.1% decrease; *p* = 0.010) (Fig. 3A), specifically in those who performed HIIT (14% reduction; *p* = 0.044) (Fig. 3B), while no change was observed in the HICT group (Fig. 3C). Among participants with T1D, no significant changes in P-selectin levels were found when data from both training modalities were pooled (Fig. 3D). However, a non-significant trend towards reduction was observed in the T1D subgroup that performed HIIT (Fig. 3E), but not in those who performed HICT (Fig. 3F).

### Correlations between inflammation and vascular adhesion markers and fitness and physical activity parameters

**Fig. 4:**
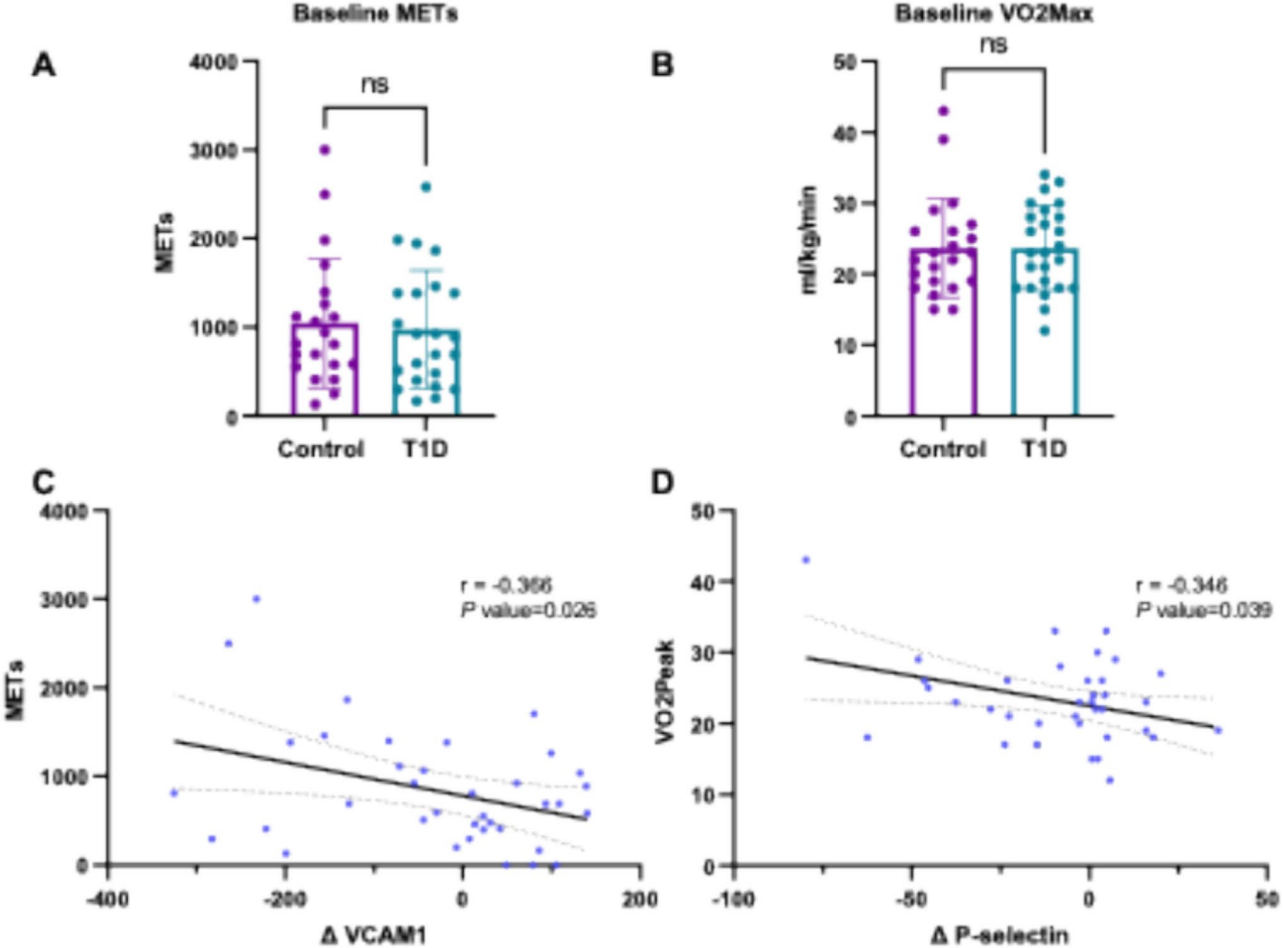
Relationship between physical activity and fitness levels at baseline and changes in adhesion molecules. **A**. Comparison between control and T1D subjects ‘ METs at baseline. **B**. VO_2_max at baseline in controls and subjects with T1D. C. Correlations between the exercise-induced changes in VCAM1 (Δ) and baseline METs. D. Correlation between exercise-induced changes in P-selectin (Δ) and baseline VO_2_max. Δ indicates post – pre values. Abbreviations: *METs* Metabolic equivalents, *VCAM1* Vascular cell adhesion molecule 1, *P-selectin* Platelet Selectin, *VO*_*2*_*max* maximal oxygen uptake. Preference for color: online only.

No significant differences were observed between groups at baseline in METs or VO₂max (Fig. 4A–B). To explore potential associations between baseline physical activity or fitness and molecular responses to training, bivariate correlation analyses were conducted across all participants, regardless of training modality or clinical status. A significant negative correlation was found between baseline METs and the change in VCAM1 levels pre- to post-intervention (*r* = –0.366; *p* = 0.026) (Fig. 4C). Additionally, a significant negative correlation was observed between baseline VO₂max and the change in P-selectin levels (*r* = –0.346; *p* = 0.039) (Fig. 4D).

## Discussion

Our results showed that patients with T1D had lower concentrations of IL-10 and a tendency toward higher concentrations of ICAM1, indicating a reduced anti-inflammatory capacity and an increased risk of atherosclerosis (Carlos and Harlan 1994). HIIT has been shown to enhance the anti-inflammatory capacity of IL-10 in patients with T1D, where this capacity typically declines as the disease progresses (dos Santos Haber et al. 2023). IL-10 is crucial for reducing the presence of antigens and the production of pro-inflammatory cytokines (Saraiva et al. 2019), and its decrease contributes to the inflammatory profile in patients with T1D, exacerbating the interaction between the immune system and pancreatic β cells (Cnop et al. 2005). The myokines released by the muscle have been shown to have an anti-inflammatory effect, and the amount of myokines released is related to the intensity of the exercise (Pedersen 2017; Scheffer and Latini 2020). However, previous studies suggest that strenuous exercise may have the opposite effect, potentially increasing inflammation (Balan and Locke 2011), indicating that the benefits of exercise depend on its modality, frequency, intensity, and duration. These findings suggest that HIIT may contribute to improving the anti-inflammatory profile in individuals with T1D, which could be of relevance in the context of disease progression. Additionally, this increase in IL-10 was observed significantly only in patients with T1D, suggesting that these exercise protocols, especially HIIT, may play a “restorative” role in the levels of this cytokine. This may explain why control subjects, who started with higher baseline IL-10 levels, did not show a significant increase after training.

 Regarding adhesion molecules, no significant differences were found between control subjects and subjects with T1D, although there was a tendency for higher ICAM1 levels. These results differ from the current literature (Gogitidze Joy et al. 2010; Iannantuoni et al. 2020), which reports that patients with T1D present higher levels of adhesion molecules such as P-selectin, VCAM1, and ICAM1. Such discrepancies could potentially be related to differences in disease management across cohorts, including factors like glycemic control or time in range, among others, which were not assessed in our study but may influence inflammatory and endothelial markers. This hypothesis is supported by previous studies showing that glycemic control and glucose variability can modulate endothelial function and the expression of adhesion molecules (Cacace et al. 2025).

Physical exercise can protect against the risk of atherosclerosis by reducing the levels of adhesion molecules VCAM1 and ICAM1 (Palmefors et al. 2014). Moreover, exercise improves blood pressure and arterial stiffness (Park et al. 2020), which are closely related to the risk of atherosclerosis. In our study, exercise in the control group reduced P-selectin and VCAM1 concentrations, with specific effects depending on the type of training. HIIT decreased P-selectin levels, while HICT tended to reduce VCAM1 levels. These results suggest that exercise intensity and/or duration may influence the regulation of these molecules, potentially aiding in the prevention of atheroma plaque formation (Krieglstein and Granger 2001).

A systematic review by Palmefors et al. (2014) summarized studies about the effects of physical activity or exercise on cytokines, chemokines, adhesion molecules, CRP or angiogenic factors, and ranked them based on the quality of evidence. Interestingly, this analysis showed that the evidence for a reduction in VCAM1 is moderate, high for ICAM1, and insufficient for P-selectin. Similar to our finding of limited reductions in adhesion molecules, Madsen et al. (2015) found no significant differences in adhesion molecules after an 8-week low volume HIIT intervention in either controls and subjects with type 2 diabetes, despite functional and structural vascular improvements (Madsen et al. 2015). Studies showing greater reductions in these molecules typically involve longer or more frequent training sessions (Adamopoulos et al. 2001; Roberts et al. 2006). This suggests that prolonged or frequent exercise protocols could lead to a general reduction in these molecule levels. Other studies that found profound effects of this type of training on inflammatory and adhesion molecules were performed in subjects with underlying pathologies involving endothelial dysfunction, such as chronic heart failure (Larsen et al. 2001; Niebauer et al. 2005). Our population with T1D consists of young, non-obese subjects with absence of any cardiovascular diseases, therefore, baseline levels of cardiovascular disease biomarkers (such as inflammatory and adhesion molecules) are not as elevated as in the aforementioned studies (Larsen et al. 2001; Niebauer et al. 2005). This could, at least in part, explain the similar levels of inflammation and adhesion molecule levels between controls and T1D and the lack of effect of the different exercise training interventions in patients with T1D.

Our study reveals that subjects’ baseline physical fitness and activity levels may be important factors determining the degree of improvement in adhesion molecule levels, which are cardiovascular disease biomarkers. Those subjects with greater baseline VO_2_max achieve greater reduction in P-selectin levels with training. In addition, in subjects that were more physically active before the training period (greater metabolic equivalent units), levels of VCAM1 were further reduced by training. In this sense, Bellos et al. (2023) showed that total physical activity, estimated as daily time spent stepping, was negatively associated with VCAM1, ICAM1 and endothelial-leukocyte adhesion molecule in a cohort of subjects with and without diabetes. Independently of adhesion molecule levels, interaction between peripheral blood mononuclear cells and endothelial cells improved after a 20-min treadmill exercise at 65–70% peak oxygen consumption in healthy subjects (Mills et al. 2006). Interestingly, this occurred only in those subjects considered fit, in terms of median VO_2_max levels, and not in less-fit subjects (Mills et al. 2006). Therefore, being physically active and having a good physical fitness seem to predispose individuals to achieve greater benefits on the vascular system.

This study has some limitations. The control and T1D groups were not strictly matched for age and sex, and there was a trend toward higher BMI and body mass in the control group, which could have influenced some of the results. Additionally, the short duration of the intervention may have limited the magnitude of detectable changes in certain biomarkers. Another limitation is the absence of C-reactive protein measurement, a widely used and sensitive marker of systemic inflammation, which could have provided complementary information to the cytokine and adhesion molecule data.

Nonetheless, our findings suggest that exercise-induced changes in circulating pro- and anti-inflammatory cytokines and CAMs may vary depending on the type of training and the population studied. Furthermore, the baseline physical activity and fitness levels appear to be associated with individual variability in response to exercise in terms of cardiovascular disease-related markers. Future studies are needed to address the specific immune cell responses and adaptations triggered by HIIT and HICT training that improve vascular health, potentially preventing cardiovascular diseases.

## Data Availability

The data presented in this study are available on request from the corresponding author.

## Abbreviations

T1D: Type 1 diabetes mellitus
JNk: C-Jun N-terminal kinase
IL-1β: Interleukin-1 beta
TNFα: Tumor necrosis factor-alpha
IL-6: Interleukin-6
CAMs: Cellular adhesion molecules
IL-10: Interleukin-10
IL-1α: Interleukin-1 alpha
NF-κB: Nuclear factor kappa B
ICAM1: Intercellular adhesion molecule 1
VCAM1: Vascular cell adhesion molecule
P-selectin: Platelet selectin
MICT: Moderate intensity continuous training
HICT: Heavy-domain Intensity Continuous Training
HIIT: High intensity interval training
PPO: Peak power output
VO_2_max: Maximal oxygen uptake
BMI: Body mass index

## References

[CR1] Adamopoulos S, Parissis J, Kroupis C, Georgiadis M, Karatzas D, Karavolias G, Koniavitou K, Coats AJS, Kremastinos DT (2001) Physical training reduces peripheral markers of inflammation in patients with chronic heart failure. Eur Heart J 22(9):791–797. 10.1053/euhj.2000.2285 11350112 10.1053/euhj.2000.2285

[CR2] Atri C, Guerfali FZ, Laouini D (2018) Role of human macrophage polarization in inflammation during infectious diseases. Int J Mol Sci 19(6):Article 6. 10.3390/ijms19061801 10.3390/ijms19061801 PMC6032107 29921749

[CR3] Balan M, Locke M (2011) Acute exercise activates myocardial nuclear factor kappa B. Cell Stress Chaperones 16(1):105–111. 10.1007/s12192-010-0217-7 20694538 10.1007/s12192-010-0217-7 PMC3024089

[CR4] Bellos I, Marinaki S, Lagiou P, Boletis IN, Stehouwer CDA, Van Greevenbroek MMJ, Eussen SJPM, De Galan BE, Savelberg HHCM, Koster A, Wesselius A, Benetou V (2023) Association of physical activity with endothelial dysfunction among adults with and without chronic kidney disease: the Maastricht Study. Atherosclerosis 383:117330. 10.1016/j.atherosclerosis.2023.117330 37837705 10.1016/j.atherosclerosis.2023.117330

[CR5] Bertoluci MC, Cé GV, Silva AMVda, Puñales MKC (2008) Disfunção endotelial no diabetes melito tipo 1. Arq Bras Endocrinol Metabol 52:416–426. 10.1590/S0004-27302008000200030 18438553 10.1590/s0004-27302008000200030

[CR6] Brugnara L, García AI, Murillo S, Ribalta J, Fernandez G, Marquez S, Rodriguez MA, Vinaixa M, Amigó N, Correig X, Kalko S, Pomes J, Novials A (2022) Muscular carnosine is a marker for cardiorespiratory fitness and cardiometabolic risk factors in men with type 1 diabetes. Eur J Appl Physiol 122(6):1429–1440. 10.1007/s00421-022-04929-z 35298695 10.1007/s00421-022-04929-z

[CR7] Cacace J, Luna-Marco C, Hermo-Argibay A, Pesantes-Somogyi C, Hernández-López OA, Pelechá-Salvador M, Bañuls C, Apostolova N, De Miguel-Rodríguez L, Morillas C, Rocha M, Rovira-Llopis S, Víctor VM (2025) Poor glycaemic control in type 2 diabetes compromises leukocyte oxygen consumption rate, OXPHOS complex content and neutrophil-endothelial interactions. Redox Biol 81:103516. 10.1016/j.redox.2025.103516 39986115 10.1016/j.redox.2025.103516 PMC11893319

[CR8] Canet F, Díaz-Pozo P, Luna-Marco C, Fernandez-Reyes M, Vezza T, Marti M, Salazar JD, Roldan I, Morillas C, Rovira-Llopis S, Rocha M, Víctor VM (2022) Mitochondrial redox impairment and enhanced autophagy in peripheral blood mononuclear cells from type 1 diabetic patients. Redox Biol 58:102551. 10.1016/j.redox.2022.102551 36455476 10.1016/j.redox.2022.102551 PMC9713367

[CR9] Carlos T, Harlan J (1994) Leukocyte-endothelial adhesion molecules. Blood 84(7):2068–2101. 10.1182/blood.V84.7.2068.2068 7522621

[CR10] Cnop M, Welsh N, Jonas J-C, Jörns A, Lenzen S, Eizirik DL (2005) Mechanisms of Pancreatic β-Cell Death in Type 1 and Type 2 Diabetes: many differences. Few Similar Diabet 54(2):S97–S107. 10.2337/diabetes.54.suppl_2.S97 10.2337/diabetes.54.suppl_2.s97 16306347

[CR11] D ‘hooge R, Hellinckx T, Van Laerthem C, Stegen S, De Schepper J, Van Aken S, Dewolf D, Calders P (2011) Influence of combined aerobic and resistance training on metabolic control, cardiovascular fitness and quality of life in adolescents with type 1 diabetes: a randomized controlled trial. Clin Rehab 25(4):349–359. 10.1177/0269215510386254 10.1177/0269215510386254 21112904

[CR12] Delmastro MM, Piganelli JD (2011) Oxidative stress and redox modulation potential in Type 1 diabetes. J Immunol Res 2011:e593863. 10.1155/2011/593863 10.1155/2011/593863 PMC3102468 21647409

[CR13] dos Santos Haber JF, Barbalho SM, Sgarbi JA, de Argollo Haber RS, de Labio RW, Laurindo LF, Chagas EFB, Payão SLM (2023) The relationship between type 1 diabetes mellitus, TNF-α, and IL-10 gene expression. Biomedicines 11(4):Article 4. 10.3390/biomedicines11041120 10.3390/biomedicines11041120 PMC10135733 37189738

[CR14] Gao C, Yue Y, Wu D, Zhang J, Zhu S (2025) Effects of high-intensity interval training versus moderate-intensity continuous training on cardiorespiratory and exercise capacity in patients with coronary artery disease: a systematic review and meta-analysis. PLoS ONE 20(2):e0314134. 10.1371/journal.pone.0314134 39977401 10.1371/journal.pone.0314134 PMC11841918

[CR15] Gogitidze Joy N, Hedrington MS, Briscoe VJ, Tate DB, Ertl AC, Davis SN (2010) Effects of acute hypoglycemia on inflammatory and pro-atherothrombotic biomarkers in individuals with type 1 diabetes and healthy individuals. Diabetes Care 33(7):1529–1535. 10.2337/dc09-0354 20587723 10.2337/dc09-0354 PMC2890354

[CR16] Heyman E, Toutain C, Delamarche P, Berthon P, Briard D, Youssef H, DeKerdanet M, Gratas-Delamarche A (2007) Exercise training and cardiovascular risk factors in type 1 diabetic adolescent girls. Pediatr Exerc Sci 19(4):408–419. 10.1123/pes.19.4.408 18089908 10.1123/pes.19.4.408

[CR17] Iannantuoni F, De Marañon A, Abad-Jiménez Z, Canet F, Díaz-Pozo P, López-Domènech S, Morillas C, Rocha M, Víctor VM (2020) Mitochondrial alterations and enhanced human leukocyte/endothelial cell interactions in type 1 diabetes. J Clin Med. 10.3390/jcm9072155 32650465 10.3390/jcm9072155 PMC7408780

[CR18] Iannetta D, Inglis EC, Mattu AT, Fontana FY, Pogliaghi S, Keir DA, Murias JM (2020) A critical evaluation of current methods for exercise prescription in women and men. Med Sci Sports Exerc 52(2):466–473. 10.1249/MSS.0000000000002147 31479001 10.1249/MSS.0000000000002147

[CR19] James S, Gallagher R, Dunbabin J, Perry L (2014) Prevalence of vascular complications and factors predictive of their development in young adults with type 1 diabetes: systematic literature review. BMC Res Notes 7(1):593. 10.1186/1756-0500-7-593 25182937 10.1186/1756-0500-7-593 PMC4167503

[CR20] Kaneto H, Matsuoka T, Nakatani Y, Kawamori D, Matsuhisa M, Yamasaki Y (2005) Oxidative stress and the JNK pathway in diabetes. Curr Diabetes Rev 1(1):65–72 18220583 10.2174/1573399052952613

[CR21] Krieglstein CF, Granger DN (2001) Adhesion molecules and their role in vascular disease*. Am J Hypertens 14(S3):44S-54S. 10.1016/S0895-7061(01)02069-6 11411765 10.1016/s0895-7061(01)02069-6

[CR22] Larsen AI, Aukrust P, Aarsland T, Dickstein K (2001) Effect of aerobic exercise training on plasma levels of tumor necrosis factor alpha in patients with heart failure. Am J Cardiol 88(7):805–808. 10.1016/S0002-9149(01)01859-8 11589856 10.1016/s0002-9149(01)01859-8

[CR23] Leick M, Azcutia V, Newton G, Luscinskas FW (2014) Leukocyte recruitment in inflammation: basic concepts and new mechanistic insights based on new models and microscopic imaging technologies. Cell Tissue Res 355(3):647–656. 10.1007/s00441-014-1809-9 24562377 10.1007/s00441-014-1809-9 PMC3994997

[CR24] Madsen SM, Thorup AC, Overgaard K, Bjerre M, Jeppesen PB (2015) Functional and structural vascular adaptations following 8 weeks of low volume high intensity interval training in lower leg of type 2 diabetes patients and individuals at high risk of metabolic syndrome. Arch Physiol Biochem 121(5):178–186. 10.3109/13813455.2015.1087033 26471849 10.3109/13813455.2015.1087033

[CR25] Maedler K, Sergeev P, Ris F, Oberholzer J, Joller-Jemelka HI, Spinas GA, Kaiser N, Halban PA, Donath MY (2002) Glucose-induced β cell production of IL-1β contributes to glucotoxicity in human pancreatic islets. J Clin Invest 110(6):851–860. 10.1172/JCI15318 12235117 10.1172/JCI15318 PMC151125

[CR26] Mills PJ, Hong S, Redwine L, Carter SM, Chiu A, Ziegler MG, Dimsdale JE, Maisel AS (2006) Physical fitness attenuates leukocyte-endothelial adhesion in response to acute exercise. J Appl Physiol 101(3):785–788. 10.1152/japplphysiol.00135.2006 16728524 10.1152/japplphysiol.00135.2006

[CR27] Murillo S, Brugnara L, Servitja J-M, Novials A (2022) High Intensity Interval Training reduces hypoglycemic events compared with continuous aerobic training in individuals with type 1 diabetes: HIIT and hypoglycemia in type 1 diabetes. Diab Metabol 48(6):6. 10.1016/j.diabet.2022.101361 10.1016/j.diabet.2022.101361 35714884

[CR28] Murillo S, Brugnara L, Ríos S, Ribas V, Servitja J-M, Novials A (2024) People with type 1 diabetes exhibit lower exercise capacity compared to a control population with similar physical activity levels. Diabetes Res Clin Pract 211:111655. 10.1016/j.diabres.2024.111655 38574895 10.1016/j.diabres.2024.111655

[CR29] Niebauer J, Clark AL, Webb-Peploe KM, Coats AJS (2005) Exercise training in chronic heart failure: Effects on pro-inflammatory markers. Eur J Heart Fail 7(2):189–193. 10.1016/j.ejheart.2004.07.012 15701465 10.1016/j.ejheart.2004.07.012

[CR30] Opazo-Díaz E, Montes-de-Oca-García A, Galán-Mercant A, Marín-Galindo A, Corral-Pérez J, Ponce-González JG (2024) Characteristics of high-intensity interval training influence anthropometrics, glycemic control, and cardiorespiratory fitness in type 2 diabetes mellitus: a systematic review and meta-analysis of randomized controlled trials. Sports Med. 10.1007/s40279-024-02114-0 39358495 10.1007/s40279-024-02114-0

[CR31] Palmefors H, DuttaRoy S, Rundqvist B, Börjesson M (2014) The effect of physical activity or exercise on key biomarkers in atherosclerosis – a systematic review. Atherosclerosis 235(1):150–161. 10.1016/j.atherosclerosis.2014.04.026 24835434 10.1016/j.atherosclerosis.2014.04.026

[CR32] Park W, Jung W-S, Hong K, Kim Y-Y, Kim S-W, Park H-Y (2020) Effects of moderate combined resistance- and aerobic-exercise for 12 weeks on body composition, cardiometabolic risk factors, blood pressure, arterial stiffness, and physical functions, among obese older men: a pilot study. Int J Environ Res Public Health 17(19):Article 19. 10.3390/ijerph17197233 10.3390/ijerph17197233 PMC7579509 33022918

[CR33] Pedersen BK (2017) Anti-inflammatory effects of exercise: role in diabetes and cardiovascular disease. Eur J Clin Invest 47(8):600–611. 10.1111/eci.12781 28722106 10.1111/eci.12781

[CR34] Petersen AMW, Pedersen BK (2005) The anti-inflammatory effect of exercise. J Appl Physiol 98(4):1154–1162. 10.1152/japplphysiol.00164.2004 15772055 10.1152/japplphysiol.00164.2004

[CR35] Riddell, M. C., Li, Z., Gal, R. L., Calhoun, P., Jacobs, P. G., Clements, M. A., Martin, C. K., Doyle Iii, F. J., Patton, S. R., Castle, J. R., Gillingham, M. B., Beck, R. W., Rickels, M. R., T1DEXI Study Group, Riddell, M. C., Rickels, M. R., Beck, R. W., Castle, J. R., Calhoun, P., … Barnes, M (2023) Examining the acute glycemic effects of different types of structured exercise sessions in type 1 diabetes in a real-world setting: the type 1 diabetes and exercise initiative (T1DEXI). Diabetes Care 46(4):704–713. 10.2337/dc22-1721 36795053 10.2337/dc22-1721 PMC10090894

[CR36] Roberts L, Jones TW, Fournier PA (2002) Exercise training and glycemic control in adolescents with poorly controlled type 1 diabetes mellitus. J Pediatr Endocrinol Metab 15(5):621–628 12014521 10.1515/jpem.2002.15.5.621

[CR37] Roberts CK, Won D, Pruthi S, Kurtovic S, Sindhu RK, Vaziri ND, Barnard RJ (2006) Effect of a short-term diet and exercise intervention on oxidative stress, inflammation, MMP-9, and monocyte chemotactic activity in men with metabolic syndrome factors. J Appl Physiol 100(5):1657–1665. 10.1152/japplphysiol.01292.2005 16357066 10.1152/japplphysiol.01292.2005

[CR38] Salem MA, AboElAsrar MA, Elbarbary NS, ElHilaly RA, Refaat YM (2010) Is exercise a therapeutic tool for improvement of cardiovascular risk factors in adolescents with type 1 diabetes mellitus? A randomised controlled trial. Diabetol Metab Syndr 2(1):47. 10.1186/1758-5996-2-47 20618996 10.1186/1758-5996-2-47 PMC3238209

[CR39] Saraiva M, Vieira P, O’Garra A (2019) Biology and therapeutic potential of interleukin-10. J Exp Med 217(1):e20190418. 10.1084/jem.20190418 10.1084/jem.20190418 PMC7037253 31611251

[CR40] Scheffer DDL, Latini A (2020) Exercise-induced immune system response: anti-inflammatory status on peripheral and central organs. Biochimica Et Biophysica Acta (BBA) - Molecular Basis of Disease 1866(10):165823. 10.1016/j.bbadis.2020.165823 32360589 10.1016/j.bbadis.2020.165823 PMC7188661

[CR41] Sharifinejad N, Zaki-Dizaji M, Sepahvandi R, Fayyaz F, dos Santos Vilela MM, ElGhazali G, Abolhassani H, Ochs HD, Azizi G (2022) The clinical, molecular, and therapeutic features of patients with IL10/IL10R deficiency: a systematic review. Clin Exp Immunol 208(3):281–291. 10.1093/cei/uxac040 35481870 10.1093/cei/uxac040 PMC9226142

[CR42] Tschakert G, Hofmann P (2013) High-intensity intermittent exercise: methodological and physiological aspects. Int J Sports Physiol Perform 8(6):600–610. 10.1123/ijspp.8.6.600 23799827 10.1123/ijspp.8.6.600

[CR43] Wu N, Bredin SSD, Guan Y, Dickinson K, Kim DD, Chua Z, Kaufman K, Warburton DER (2019) Cardiovascular health benefits of exercise training in persons living with type 1 diabetes: a systematic review and meta-analysis. J Clin Med 8(2):253. 10.3390/jcm8020253 30781593 10.3390/jcm8020253 PMC6406966

[CR44] Yang J, Cao RY, Gao R, Mi Q, Dai Q, Zhu F (2017) Physical Exercise Is a Potential “Medicine” for Atherosclerosis. In: Xiao J (ed) Exercise for Cardiovascular Disease Prevention and Treatment From Molecular to Clinical, Part 1. Springer, Berlin, pp 269–286 10.1007/978-981-10-4307-9_15 29022268

[CR45] Yang D-R, Wang M-Y, Zhang C-L, Wang Y (2024) Endothelial dysfunction in vascular complications of diabetes: a comprehensive review of mechanisms and implications. Front Endocrinol 15:1359255. 10.3389/fendo.2024.1359255 10.3389/fendo.2024.1359255 PMC11026568 38645427

